# Incidence of and risk factors for hip fracture in Nagasaki, Japan from 2005 to 2014

**DOI:** 10.1007/s11657-021-00978-7

**Published:** 2021-07-10

**Authors:** Hironobu Koseki, Shinya Sunagawa, Chieko Noguchi, Akihiko Yonekura, Umi Matsumura, Kaho Watanabe, Yuta Nishiyama, Makoto Osaki

**Affiliations:** 1 Department of Health Sciences, Nagasaki University Graduate School of Biomedical Sciences, 1-7-1 Sakamoto, Nagasaki, 852-8520 Japan; 2 Department of Rehabilitation, Wajinkai Hospital, 96 Nakazato, Nagasaki, 851-0103 Japan; 3 Department of Orthopedic Surgery, Nagasaki University Graduate School of Biomedical Sciences, 1-7-1 Sakamoto, Nagasaki, 852-8501 Japan

**Keywords:** Hip fracture, Incidence, Japan, Nagasaki, Risk factor

## Abstract

**Summary:**

The annual incidence of new hip fractures increased from 2005 to 2014 in Nagasaki and females were much more affected. High-risk factors were identified as age ≥ 80 years, winter, indoors, living room, Monday, and early morning. Seven days after admission, most patients remained hospitalized and had been treated surgically.

**Introduction:**

Hip fractures are major osteoporotic fractures that reduce quality of life. In Japan, the incidence of hip fractures increased steadily from 1986 to 2014 and the number of hip fractures could be 7.3–21.3 million by 2050. This study aimed to determine the incidence of hip fractures from 2005 to 2014 in Nagasaki Prefecture and to analyze the characteristics of and risk factors for hip fracture.

**Methods:**

Hip fractures that occurred in Nagasaki Prefecture between 2005 and 2014 were analyzed using emergency transportation records. Fracture type, age, sex, location in which fracture occurred, and risk factors for hip fracture were clarified.

**Results:**

The total number of new hip fractures among individuals ≥ 35 years old was 17,395 (mean age, 82.6 years old) and the annual incidence per 100,000 population increased from 147.9 in 2005 to 235.0 in 2014. Females (79.6%) were much more commonly affected than males (20.4%) and cervical fractures were more common than trochanteric fractures in all age groups. Hip fracture tended to be associated with age ≥ 80 years, winter rather than summer, indoors rather than outdoors, and living room rather than the bathroom or toilet. Other high-risk factors were Monday as day of the week, and early morning as the time of day. Seven days after admission, 97.3% of patients were hospitalized and 78.1% of hip fractures had been treated surgically.

**Conclusion:**

Information on actual situations and valid preventive measures relevant to hip fracture are urgently needed.

## Introduction

Hip fracture is a serious complication of osteoporosis and represents a major problem worldwide. The burden of osteoporosis in patients with hip fracture is now well recognized, involving economic costs as well as morbidity and premature mortality. The incidence of hip fracture increases along with the population of elderly individuals, since hip fracture incidence increases exponentially with age. Numbers of hip fractures worldwide for males and females in 1990 were estimated as 338,000 and 917,000, respectively, with totals expected to approximately double from 1.26 to 2.6 million by 2025, and to increase to 7.3–21.3 million by 2050 [1]. In 1990, 26% of all hip fractures occurred in Asia. By 2050, more than 50% of hip fractures are projected to occur in Asia [2]. The total population of Japan is predicted to keep decreasing until 2050, along with a substantial increase in the proportion ≥ 85 years old. The annual number of patients with new hip fractures in Japan was thus estimated to be approximately 175,700 in 2012 and 320,000 in 2040 based on previously reported incidences [3, 4]. The Japanese Orthopaedic Association (JOA) performed nationwide surveys of hip fracture annually from 1998 to 2014 to elucidate the current status of hip fractures in Japan. These surveys found a drastic increase in the number of patients, particularly among females. However, most epidemiological studies investigating the incidence of hip fracture have been based on data tallied from general surveys within a limited response rate and by individual hospitals except for non-affiliated institutions, and thus do not entirely reflect the actual situation in specific regions. Moreover, risk factors for hip fracture have been analyzed in smaller numbers of cases, based on the contents of medical records or interviews surveying individual elderly patients.

In Japan, all ambulance services are required by law to record prehospital transport data, such as age, sex, address, and times of emergency call and hospital arrival. In particular, Nagasaki Prefecture has maintained original emergency transportation records since 2004, including additional information such as diagnostic codes, definitive diagnosis, and outcome at 1 week after ambulance transport. Thirteen main diagnostic codes are used by the treating physicians. Coding of injuries due to external causes is divided into the following: traumatic intracranial hemorrhage; cardiovascular and lung injury; abdominal organ injury; pelvic fracture; other fractures; severe multiple trauma; spinal cord injury; asphyxia; burn; drowning; poisoning; minor injuries; and proximal femoral fracture (hip fracture). In Nagasaki Prefecture, all ambulance attendants must submit half of an emergency transportation record with prehospital data, then hospitals (attending physicians) are encouraged to submit the other half of the record with the definitive diagnosis and outcome at 1 week after ambulance transport. The participation rate of ambulance attendants is 100% and the average collection rate from hospitals after 1 week has been reported as 91.6% [5]. This high collection rate of emergency transportation records offers reliable objective data and enables high-quality analysis of severe disease and trauma in a specific region. The present study had three aims: to elucidate the status of hip fracture in Nagasaki Prefecture from 2005 to 2014; to survey outcomes at 1 week after ambulance transport; and to investigate the causes and risk factors of hip fracture.

## Materials and methods

### Study design and data

We conducted a retrospective study using standardized regional data from emergency transportation records collected in the Nagasaki Medical Control Council. From April 2004 to March 2015, a total of 522,912 patients were transported by ambulance in Nagasaki Prefecture and the mean collection rate of emergency transportation records was 93.1% (*n* = 486,852). Inclusion criteria for this study were as follows: fracture of the femoral neck (cervical or trochanteric) as a definitive diagnosis; and age ≥ 35 years. The definitive diagnosis was made by the attending physician at 1 week after ambulance. We excluded cases with no definitive diagnosis, no description of outcome, or unknown type of accident in the emergency transportation records. Patients transferred from another hospital and those with unknown transport type were also excluded.

The parameters to be analyzed included ambulation date and time, sex (male or female), age (5-year strata), transport time, street address, destination hospital, affected side, fracture type (cervical or trochanteric), area in which the fracture occurred (indoors vs. outdoors), and outcomes at 1 week after ambulance transport, including status, transferred hospital, and treatment method. The incidence of hip fractures in each study year was calculated as the number of fractures per 100,000 population per year. The initials and date of birth were omitted from database information to ensure protection of personal information. Duplication of cases was therefore checked based on ambulance number, date, and time of ambulation. Area in which the injury occurred was categorized as “house,” “outdoors,” “hospital,” “facilities for the elderly,” and “public.” Status at 1 week after ambulance transport was divided into five categories: “hospitalization”; “transfer to a different hospital”; “discharge from the hospital in an ambulatory state”; “mortality”; and “unknown.” The present study was approved by the research ethics committee at Nagasaki University Graduate School of Biomedical Sciences (approval number 16031085).

### Statistical analysis

Significant differences between two groups were tested with Mann–Whitney U tests for unpaired values (for the number of patients by sex, fractured side, and fracture type). The categories/ratios of variables such as outcomes and treatment history at 1 week after ambulance transportation were compared using Pearson’s chi-squared test. Continuous variables such as age strata, seasonal, and daily variations were tested with one-way analysis of variance multiple comparison tests followed by post hoc Tukey–Kramer and Bonferroni-Dunn multiple comparison tests. All data were analyzed using SPSS version 22.0 (SPSS, Chicago, IL, USA). Values are expressed as mean ± standard deviation. Statistical significance was defined for values of *P* < 0.01.

## Results

### Number and incident rate of patients

The total number of new hip fractures among individuals ≥ 35 years old was 17,395 and the mean age was 82.6 ± 9.4 years. The total number of patients and population ≥ 35 years old in Nagasaki Prefecture in each year are summarized in Table 1. The actual number of new hip fractures increased, while the population decreased year after year. As a result, the incidence tended to rise gradually from 147.9 per 100,000 population per year in 2005 to 235.0 per 100,000 population per year in 2014.

**Table 1:** Total number of hip fractures, relevant populations, and fracture type-, sex-, and affected side-specific patient totals from 2005 to 2014

Year	Total population	Population aged ≥ 35 years	Hip fractures aged ≥ 35 years	Incidence per 100,000	Cervical/trochanteric^*^	Cervical/trochanteric Ratio	Male/female^†^	Rt/Lt^#^
2005	1,478,632	941,412	1,392	147.9	819/377	2.17	275/1009	629/654
2006	1,466,512	943,288	1,523	161.5	829/513	1.62	305/1120	708/701
2007	1,453,740	944,963	1,511	159.9	876/462	1.90	310/1125	660/679
2008	1,441,451	946,649	1,601	169.1	864/546	1.58	326/1197	729/735
2009	1,432,236	949,143	1,696	178.7	867/645	1.34	338/1309	728/750
2010	1,426,779	950,415	1,775	186.8	940/787	1.19	330/1377	821/846
2011	1,417,282	950,575	1,964	206.6	984/814	1.21	342/1567	882/946
2012	1,407,925	950,077	1,718	180.8	953/730	1.31	341/1316	762/804
2013	1,396,481	948,066	1,993	210.2	990/810	1.22	391/1539	892/932
2014	1,385,533	945,719	2,222	235.0	1148/909	1.26	453/1736	1042/987

^*^Excluding 1532 unknown cases

^†^Excluding 689 unknown cases

^#^Excluding 1508 unknown cases

### Fracture type-, sex-, and affected side-specific patient totals

A total of 9,270 cervical fractures (mean age; 82.1 ± 9.4 years), 6,593 trochanteric fractures (mean age; 84.1 ± 8.7 years), and 1,532 unclassified fractures were detected and changes over time in the prevalences of both fracture types are shown in Table 1. Although cervical fractures were significantly more common than trochanteric fractures during the observation period (*P* < 0.01), the cervical/trochanteric ratio decreased over time, meaning that the growth rate of trochanteric fractures was higher than that of cervical fractures. There were almost 3.9 times more females with hip fractures (79.6%) than males (20.4%), and the ratios of females with cervical and trochanteric fractures were 79.4% (7,082 cases) and 80.5% (5,112 cases), respectively. The affected side in total was 7,853 cases (49.4%) on the right side and 8,034 cases (50.6%) on the left side, and ratios of the affected side remained almost unchanged during the observation period (*P* > 0.01).

### Age- and sex-specific patient totals

**Fig. 1:**
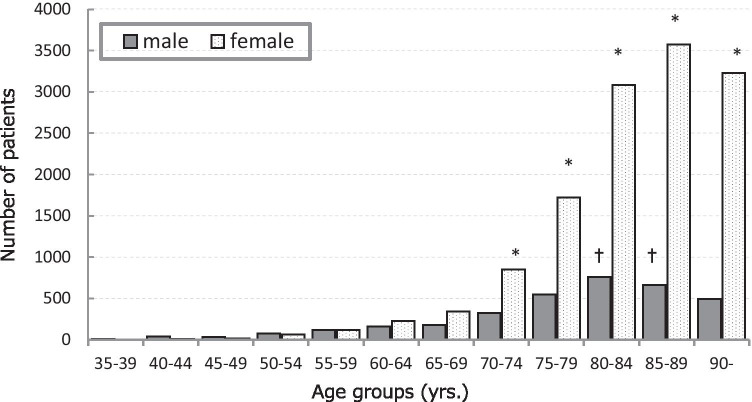
Total age- and sex-specific numbers of patients with hip fractures. The number of fractures increases sharply after 75 years old. Patients ≥ 80 years old account for 70.9% of the total number of patients and these increases are even more prominent for female patients. ^*^P < 0.01 compared with 35- to 65-year-old age groups. ^†^*P* < 0.01 compared with 35- to 75-year-old and 90-year-old age groups

Total age- and sex-specific numbers of patients were divided into 12 strata in 5-year intervals, as shown in Fig. 1. Most hip fractures occurred in patients ≥ 80 years old, comprising 11,805 cases and representing 70.9% of the total. The 80- to 85-year-old age stratum in males and the 70- to 90-year-old stratum in females showed a significantly higher number of fractures than the 35- to 65-year-old age groups (*P* < 0.01). The transition of age-specific patients from 2005 to 2014 revealed a gentle increase in the number of patients ≥ 80 years old, and a more prominent increase for patients ≥ 90 years old.

### Monthly and daily variations

**Fig. 2:**
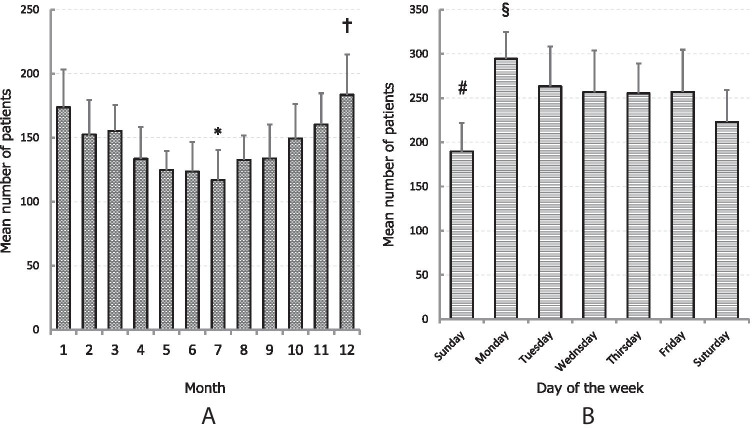
Variation in hip fracture occurrence. **A** Months of the year. **B** Days of the week. Hip fracture tends to occur more frequently in winter than in summer, and more frequently on Monday and not as frequently on Sunday compared to other days of the week. ^*^P < 0.01 compared with November, December, and January. ^†^*P* < 0.01 compared with April to September. ^#^*P* < 0.01 compared with Monday through Fridayy. ^§^*P* < 0.01 compared with Saturday and Sunday

The total number of patients per month was highest in December and lowest in July in every year from 2005 to 2014 (Fig. 2A). Mean numbers were significantly higher in December than in April, May, June, July, August, or September, and numbers were significantly lower in July than in November, December, or January (*P* < 0.01). Daily variations are shown in Fig. 2B. Incidence of hip fracture was significantly higher on Mondays than on Saturdays or Sundays, and fractures tended to occur less frequently on Sundays than on weekdays (Monday through Friday) (*P* < 0.01).

### Areas and places of injury

The number and percentage of areas and places in which hip fractures occurred are summarized in Table 2. Hip fracture occurred more frequently indoors (house, 58.3%; hospital, 20.6%; facilities for the elderly (nursing home, 9.2%; group home, 2.4%)) than outdoors (public: 9.1%). In the house, the living room (76.4%) and corridor (15.0%) were more common locations for hip fracture than the toilet or bathroom.

**Table 2:** Differences in area and room of the house in which injury occurred

Area of injury (n = 3643)	n (%)	Place of injury in the house (n = 815)	n (%)
House	2,125 (58.3)	Living	623 (76.4)
Hospital	750 (20.6)	Corridor	122 (15.0)
Nursing home	336 (9.2)	Garden	20 (2.5)
Public	332 (9.1)	Kitchen	18 (2.2)
Group home	87 (2.4)	Stairs	9 (1.1)
Not indicated	13 (0.4)	Toilet	9 (1.1)
		Bathroom	5 (0.6)
		Unknown	9 (1.1)

### Variations in time of day

**Fig. 3:**
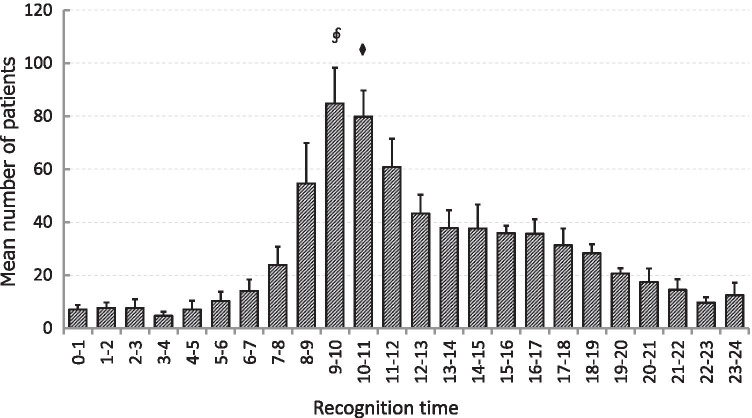
Variation in hip fracture occurrence between hours of the day. Hip fracture tends to occur more frequently at 09:00–11:00 compared with other timeframes. ^§^*P* < 0.01 compared with other timeframes except 10:00–11:00. ^⧫^*P* < 0.01 compared with other timeframes except 09:00–10:00

Recognition time (the time at which the ambulance was called) was divided into 24 timeframes and the mean number of patients per year was distributed as shown in Fig. 3. The number of ambulance calls involving hip fracture was greater during the day, particularly in the morning, and was lowest at midnight. The number of patients peaked from 09:00 to 10:00 (*P* < 0.01).

### Outcomes and treatments at 1 week after ambulance transport

At 1 week after ambulance transport, 83.7% of patients with hip fracture remained at the hospital where they had first been brought, much more than the proportions of transfers (13.6%), mortality (0.43%), or discharge from the hospital in an ambulatory state (0.06%) (Table 3). In total, 78.1% of patients had been treated surgically within 1 week after ambulance transport. The remaining 21.9% of patients were thought to be awaiting surgery (preoperative) or did not meet the indications for surgery. The rate of surgical treatment within 1 week was higher for trochanteric fracture (82.9%) than for cervical fracture (74.6%).

**Table 3:** Outcomes and treatment history at 1 week after ambulance transportation for cervical and trochanteric fractures

	Cervical n (%)	Trochanteric n (%)	Total n (%)	*P*-value
Outcome 1 week after ambulance (n = 15,806)				
Hospitalization	7,526 (81.5)	5,697 (86.7)	13,223 (83.7)	< 0.01
Transfer	1,442 (15.6)	708 (10.8)	2,150 (13.6)	< 0.01
Ambulatory	8 (0.09)	2 (0.03)	10 (0.06)	0.166
Mortality	42 (0.45)	26 (0.40)	68 (0.43)	0.576
Unknown	218 (2.36)	137 (2.09)	355 (2.25)	0.250
Total	9,236 (100)	6,570 (100)	15,806 (100)	
Treatment 1 week after ambulance (n = 14,791)				
Surgery	6,362 (74.6)	5,190 (82.9)	11,552 (78.1)	< 0.01
Conservative	2,171 (25.4)	1,068 (17.1)	3,239 (21.9)	< 0.01
Total	8,533 (100)	6,258 (100)	14,791 (100)	

## Discussion

The frequency of hip fracture appears to be increasing in many countries, particularly in Asia. Indeed, previous secular trends with modest assumptions have estimated numbers of 7.3 to 21.3 million by 2050 [1]. In Japan, the proportion of elderly individuals > 65 years old exceeded 28.0% in 2019 and is estimated to increase to 36.6 million by 2025 [4, 6], representing the highest percentage in the world since 2005. In addition, the incidence of osteoporotic hip fractures increased steadily from 1986 to 2014 in Japan [3, 4]. Nagasaki Prefecture, with an area of about 4,131 km^2^ and a population of about 1,325,000 in 2019, is located in the southwestern part of Japan. The population of residents > 65 years old has been increasing and reached 427,988 in 2019, accounting for 32.5% of the total population. Nagasaki Prefecture can thus be regarded as a model of future population structures for developed countries, including Japan.

This study revealed the actual number and incidence of hip fractures in Nagasaki Prefecture from 2005 to 2014. A major strength of this study was that we analyzed large-scale data from original emergency transportation records, which have a high collection rate (93.1%), and include additional information at 1 week after ambulance transport. This methodology can supply reliable, objective data, and enable high-quality analysis within a specific region. As a result, the annual number (1392–2222 cases) and incidence (147.9–235.0 per 100,000) of new hip fractures among the population ≥ 35 years old increased gradually from 2005 to 2014. Numbers in males and females have been increasing in a similar manner, mainly ascribed to the increase in the elder population. Life expectancy over the age of 50 years has increased, but elderly individuals experience increased fragility of bone and decreased motor function [7]. The incident rate in this study was slightly lower compared with previous nation- and worldwide survey studies [1, 4, 7, 8]. The main factors influencing differences in results were thought to be differences in research methodology, as we calculated and analyzed data from emergency transportation records. Therefore, cases brought to the hospital by means other than ambulance transport were not counted in our data. However, most patients with hip fracture could not move without help from others, so the incidence in this study was thought to approximate the number of hip fractures that occurred in a limited region and calculation of the incidence for the population living in the area was appropriate. Thus, the genuine incidence rate was thought to be somewhere between that in the present study and the results of previous survey studies. Moreover, the tendency toward annual increases in hip fracture was consistent with findings from previous studies [4, 7, 8], and the incidence of hip fracture was therefore estimated to continue at a high rate for a while in Japan.

Fracture type- and affected side-specific analyses revealed that cervical fractures (58.4%) occurred more frequently than trochanteric fractures (41.6%) among patients > 35 years old, while the affected side was similar between right and left. In Japan, trochanteric fractures have been reported to be more common than cervical fractures [9, 10]. Although the exact reason cervical fractures were more common than trochanteric fractures in Nagasaki Prefecture remains unknown, the possibility that physicians other than orthopedic surgeons might misdiagnose trochanteric fracture as “cervical fracture” could not be excluded. The results for the affected side were consistent with the findings of Horii et al. [9].

Age- and sex-specific data indicated that almost 3.9 times more females (79.6%) were affected than males (20.4%), and most hip fractures occurred in patients ≥ 80 years old (70.9%) of the total. The 80- to 85-year-old age stratum in males and 70- to 90-year-old age stratum in females contained more cases than 35- to 65-year-old age groups. Orimo et al. [4] and Hagino et al. [11] reported that the number of female patients was about 3.6–3.7 times higher than that of male patients, similar to our results. One reason was speculated to be the longer life expectancy of females (female: 86.83 years; male: 80.50 years in 2014 in Japan [12]). Furthermore, the incidence of hip fracture is well known to increase exponentially over 80 years old. In this study, more cervical fractures than trochanteric fractures occurred among patients over 35 years old, and as age increased, the cervical/trochanteric ratio decreased. Because trochanteric fractures show a closer relationship to low bone mass than cervical fractures [13], the percentage of trochanteric fractures enlarged among the more elderly population. Horii et al. [9] reported the mean age of patients with hip fracture in Kyoto was 82.4 ± 7.4 years for cervical fracture and 85.0 ± 7.0 years for trochanteric fracture, slightly lower than those in the present study, particularly for cervical fracture. This may be due to differences in aging rates (population > 65 years old/total population) in each region (Kyoto, 26.9%; Nagasaki, 28.9% in 2014) [14].

Several studies have demonstrated characteristic seasonality in hip fractures [10, 15–17]. The present study clearly demonstrated an increase in the number of patients with hip fracture in winter, and a decrease in summer, consistent with the findings of Hagino et al. [11]. Possible reasons have been suggested to include low blood pressure caused by low ambient temperature, a lack of vitamin D during the winter season exacerbating bone fragility, and decreased muscle strength, which increases the risk of falls [16, 18]. Daily variations in hip fracture have not been investigated previously. The present study revealed that hip fractures in Nagasaki tended to occur on Mondays, rather than on weekends. The reasons for this remain unclear. Taking into consideration that most hip fractures occurred at home, one possibility is that family members may be more attentively watching and caring for elderly individuals on weekends, resulting in a lower risk of fracture.

Focusing on the area and location of injury, 88.1% of patients with hip fracture were injured indoors, in the house, hospital, or nursing home. Furthermore, 76.4% of fractures occurring indoors were in the living room rather than the bathroom or toilet. Rates of injuries resulting from simple falls indoors are reportedly higher in older age groups [9–11]. Considering the results for seasonality, another explanation may be that elderly individuals may easily fall by catching a foot on slippers, the power cord of heating appliances, carpet, or thick bedclothes. In addition, setting up obstructive fall-prevention aids in the living room tends to be greatly disliked. Tidying up and paying attention to the floor of living rooms may thus be worth emphasizing as a measure to prevent simple falls and hip fractures.

This study appears to be the first to report variations in hip fracture by time of day. A certain proportion of cases are likely to have involved family members or other caregivers calling the ambulance after finding the patient with hip fracture, especially for elderly individuals living alone. However, most hip fractures occur by falls from a standing or sitting position, and injury rates in bed reportedly comprise only 1.0–2.2% of cases [8–10]. Recognition time recorded by the ambulance was thus regarded as the best available proxy for the time at which the injury actually occurred in this study. Hip fracture occurred more often in the daytime than during the night, and in the morning more often than in the evening, particularly within the period of 09:00–11:00. This result indicates that the morning when elderly individuals begin to move and act represents a high risk for falls and hip fracture. Seniors with decreased physical activity thus need to be careful while move after waking up and acting in the morning, especially on cold days.

Generally, hip fractures are treated surgically, except in special cases where surgical treatment is not needed or cannot be performed. In addition, early surgery (within 2 days after injury) is recommended to minimize postoperative hospitalization time and complications [19, 20]. Accordingly, 86,000 hip fractures annually are treated with surgery in Japan [10, 11] and the treatment cost for hip fracture in the UK has been estimated at £12,163 per person, including all fees during the hospitalization period [21]. The present study found that 97.3% of patients were still hospitalized (83.7% in the receiving hospital and 13.6% transferred to another hospital), and surgical treatment had been performed in 78.1% within 1 week after ambulance transport. The remaining 21.9% of patients included cases still scheduled for surgery and those outside of the surgical indication due to poor general status or comorbidities. Waiting times for surgery have been shortened in recent years, but are still relatively long in Japan compared with other countries [11, 22, 23]. Causes for delays in surgery have been reported as difficulty in using operating rooms, preparations for implants, and addressing comorbidities and the use of anticoagulant agents [8]. To shorten the length of hospitalization before surgery, sufficient cooperation among orthopedic surgeons, the facility, anesthesiologists, and internists is essential.

The mortality rate of 0.43% in this study was extraordinarily low compared to previous epidemiological research. Stenqvist et al. [24] and Ogawa et al. [17] reported overall in-hospital mortality rates of 6.3% and 1.2%, respectively. Although detailed reasons for our result remain unclear, this inconsistency might be partially attributable to the relatively short observation period in our study, compared to the mean lengths of hospitalization of 24.9 days [24] and 35.96 days [17], respectively, in those previous investigations. In this study, mortality more than 8 days after ambulance transport or after transfer to other institutions was not able to be obtained and included. Meanwhile, Tsai et al. [25] reported an in-hospital mortality rate of 6.7% within an average of 6.65 days of hospitalization. Some differences in patient demographics and data collection methods existed between the work by Tsai et al. [25] and our study. Their data were collected from the United States National Trauma Data Bank registry, and data collection was limited to relatively severe patients over 65 years old with some kind of comorbid illness or secondary fractures due to high-energy trauma.

Several limitations to this study must be considered. First, we investigated data from emergency transportation records in this study, so patients injured within the same institution or admitted to the hospital by other means could not be counted. Second, even though a definitive diagnosis was written in the records as hip fracture, 10 cases (0.06%) were in the category of “discharge from the hospital in an ambulatory state” 1 week after ambulance transport. The exact reasons for this were unknown. Although these limitations could cause some bias, the relatively large number of patients collected in this study spanning one decade may compensate for such deficits.

## Conclusions

The present study investigated a total of 17,395 cases of hip fracture among individuals ≥ 35 years old from 1986 to 2014 in Nagasaki Prefecture using emergency transportation records and analyzed the current incidence, trends, characteristics, and risk factors for such fractures. The mean age at injury was 82.6 years and the annual incidence per 100,000 population increased from 147.9 in 2005 to 235.0 in 2014. Females (79.6%) were much more commonly affected than males (20.4%) and cervical fractures were more common in all age groups. Risk factors identified included age ≥ 80 years, winter season, indoor areas, living room as location in the house, Monday as the day of the week, and early morning as the timeframe. At 7 days after admission, 97.3% of patients remained hospitalized, and 78.1% of hip fractures had been treated surgically.

## Data Availability

The authors do not wish to share the data because the dataset is part of ongoing study protocols.
